# Knockdown of LINC01614 inhibits lung adenocarcinoma cell progression by up‐regulating miR‐217 and down‐regulating *FOXP1*

**DOI:** 10.1111/jcmm.13483

**Published:** 2018-06-22

**Authors:** Ai‐Na Liu, Hua‐Jun Qu, Cai‐Yan Yu, Ping Sun

**Affiliations:** ^1^ Department of Medical Oncology the Affiliated Yantai Yuhuangding Hospital of Qingdao University Medical College Yantai Shandong China

**Keywords:** lung adenocarcinoma, LINC01614, miR‐217, *FOXP1*

## Abstract

We tried to identify the function of LINC01614 in lung adenocarcinoma (LUAD) and reveal its underlying mechanisms. qRT‐PCR was applied to assess the expression of LINC016014 in LUAD tissues, noncancerous tissues and cells. Through colony formation assay, MTT assay and apoptosis analysis, we examined the variation of cell proliferation and apoptosis ability after silencing LINC01614. Moreover, the targeting interactions among LINC01614, miR‐217 and *FOXP1* were validated *via* luciferase reporter assay, and then, we regulated the expression of miR‐217 and *FOXP1* to ascertain their importance in cell proliferation and apoptosis. LINC01614 and *FOXP1* were found to be up‐regulated in LUAD tumours and cells, whereas miR‐217 was down‐regulated. The experiment showed that target‐specific selectivity exists between LINC01614‐miR‐217 and miR‐217‐*FOXP1* 3′UTR. Furthermore, we disclosed that inhibition of LINC01614 could activate miR‐217, which subsequently restrained *FOXP1*. It was proved that LINC01614 promoted *FOXP1* by inhibiting miR‐217, which ultimately stimulated the development of LUAD.

## Introduction

Lung cancer, which consisted of non‐small cell lung cancer (NSCLC) and small cell lung cancer (SCLC), maintains a high mortality rate among a variety of cancers 1. NSCLC includes lung squamous cell carcinomas, LUADs and large cell carcinomas 1. LUAD is the most prevalent subtype of NSCLC, with an increasing morbidity and mortality rate over these years worldwide. Platinum‐based regimens are standard chemotherapies, but its side effects usually weakened the therapeutic effects. Therefore, LUAD patients still have very low survival rate in spite of multiple therapies 2. It is urgent for researchers to identify more efficient and effective therapeutic targets for LUAD 3.

Long non‐coding RNAs (lncRNAs) represent an emerging and fascinating class of transcripts, which play a significant role in many different bioprocesses 4. For instance, by binding to miRNA‐binding sites, lncRNA functions as the regulator of gene expressions and this kind of regulation has been detected in various diseases 5, 6, 7, 8, 9. LncRNA could be effective biomarkers for early diagnosis and screening of cancer in specific tissues and organisms 10. Increasing number of studies about lncRNA relating to various cancers, including colon cancer, oesophageal squamous cell carcinoma and gastric cancer, have been intensively conducted 11, 12, 13. Several lncRNAs related to lung cancer have been discovered, *for example* MALAT1 14, LINC01186 15 and LINC01207 16. With the rapid development of cutting‐edge techniques like RNA sequencing and microarray, more and more lncRNAs in lung cancer have been characterized 17, 18. Located in the genomic 2q35 locus, LINC01614 has two exons and could be transcribed into an lncRNA of 2904 nucleotides (nt). In the current research, LINC01614 expressions and functions were closely investigated.

MicroRNAs (miRNAs) are small interference RNAs with about 20nt which could regulate gene expressions *via* binding to mRNA and silencing target genes 19. Mounting evidence has indicated that miRNAs participate in many important bioprocesses, including cell proliferation, differentiation and apoptosis 20, 21. Down‐regulation of miR‐217 is frequently discovered in various cancers, suggesting its important role in tumorigenesis 22, 23, 24. However, the potential role of miR‐217 in LUAD is elusive. In this study, we studied miR‐217 expressions in LUAD tissues and cell lines and its effects on morphologic changes in LUAD.

The forkhead box P subfamily is a group of transcription factors with highly conserved forkhead or winged helix DNA‐binding domain 25, 26, which take part in a wide range of developmental events, including cellular differentiation and proliferation, pattern formation, immune regulation, signal transduction and oncogenesis 27. Recent researches have revealed that some members of this protein family are relevant to tumorigenesis and cancer progression, and an increasing amount of evidence suggests that *FOX* can act as direct targets and indirect effectors of therapeutic intervention. For example, *FOXP1* dysregulation is related to poor prognosis in B‐cell lymphomas and NSCLC 28, 29. However, when it comes to other cancers like neuroblastoma or prostate cancer, *FOXP1* can inhibit cell growth and attenuate tumorigenesis as a tumour suppressor 30, 31. Hence, the role of *FOXP1* in tumour progression is uncertain.

Here, we demonstrated LINC01614 played an important role in LUAD. The results showed that LINC01614 strongly promoted tumorigenesis and progression through miR‐217/*FOXP1* axis. This study may offer new knowledge for understanding the occurrence and development of LUAD.

## Materials and methods

### Clinical samples

A total of 23 pairs of LUAD tissues and matched normal tissues were obtained from the Affiliated Yantai Yuhuangding Hospital of Qingdao University Medical College; all of them were independently diagnosed by at least three experienced pathologists. All patients never receive chemoradiotherapy or other treatments before operation. All samples were immediately transferred to frozen liquid nitrogen and stored in −80°C refrigerator before further analysis. Each participant signed informed consent, and this study went through the ethic approval of Ethics Committee of the Affiliated Yantai Yuhuangding Hospital of Qingdao University Medical College.

### Cell culture

Human normal bronchial epithelium cells (BEAS‐2B) and LUAD cells (NCI‐H1395 and NCI1975) were purchased from the Cell Bank of the Chinese Academy of Sciences, Shanghai, China. BEAS‐2B cells were incubated in LHC‐9 medium (Biofluids, Inc., Rockville, MD, USA), which contained the following materials: 0.5 ng/ml epidermal growth factor, 500 ng/ml hydrocortisone, 0.035 ng/ml bovine pituitary extract, 500 nM phosphoryl ethanolamine, 500 nM ethanolamine, 0.01 mg/ml epinephrine, 0.1 ng/ml retinoic acid and micronutrients, while NCI‐H1395 and NCI‐H1975 cells were incubated in RPMI‐1640 medium (GIBCO BRL, Grand Island, NY, USA) supplement with 1.5 g/l NaHCO_3_, 10% FBS, 2.5 g/l glucose and 0.11 g/l sodium pyruvate. Cells were maintained at 37°C in a damp incubator, which supplemented with 5% CO_2_.

### Quantitative RT‐PCR

After being isolated by TRIzol reagent (Invitrogen, Carlsbad, CA, USA) and quantified by NanoDrop 2000 (Thermo Fisher Scientific Inc, Rockford, IL, USA), 20 ng of total RNA underwent reverse transcription by ReverTra Ace qRT‐PCR Kit (Toyobo, Osaka, Japan) on the basis of the manufacturer's instruction, and the product was then used for real‐time PCR by THUNDERBIRD SYBR^®^ qPCR Mix (Toyobo). PCR conducted as follows: predegeneration at 94°C for 2 min.; denaturation at 94°C for 30 sec.; annealing at 56°C for 30 sec.; extension at 72°C for 1 min.; repeating the previous process for 30 times; continuous extension at 72°C for 10 min. With GRAPDH and U6 as the internal references, experiment of each group was repeated three times, respectively, and quantification of the relative expression levels of lncRNA, mRNA and miRNA by $2^{-\Delta \Delta \;C_{t}}$ method. Primers sequences were present in Table 1.

**Table 1:** Primer sequences for qRT‐PCR

	Primer sequences
LINC01614‐F	5′‐CAACCAAGAGCGAAGCCAAG‐3′
LINC01614‐R	5′‐CGCCCCAAAACAACTGAGTC‐3′
*FOXP1*‐F	5′‐CCAGGCAGCTTCATGATGTG‐3′
*FOXP1*‐R	5′‐CAGCAACCACTCAGTTTAGAAGCA‐3′
miR‐217‐F	5′‐CGCAGATACTGCATCAGGAA‐3′
miR‐217‐R	5′‐CTGAAGGCAATGCATTAGGAACT‐3′
β*‐actin*‐F	5′‐CTGGAACGGTGAAGGTGACA‐3′
β*‐actin*‐R	5′‐CGGCCACATTGTGAACTTTG‐3′
U6‐F	5′‐CTCGCTTCGGCAGCACA‐3′
U6‐R	5′‐AACGCTTCACGAATTTGCGT‐3′

F, Forward; R, Reverse.

### Western blot

Total protein was lysed with RIPA lysate (Beyotime, Shanghai, China). After being quantified by Pierce BCA Protein Assay Kit (Pierce, Rockford, IL, USA), 10 μg of proteins was electrophoresed in SDS‐PAGE and blotted on a PVDF membrane for 120 min. under 200 mA constant current. The membrane was blocked in Tris buffer saline‐Tween‐20 (TBST) with 5% fat‐free milk at room temperature for 1 hr. Then, primary antibody (anti‐FOXP1, ab16645, 1:5000; anti‐GAPDH, ab9485, 1:2500; Abcam, Cambridge, MA, USA) was incubated overnight under 4°C. Then, the membrane was rinsed in TBST for 3 times. Afterwards, the secondary antibody (goat anti‐rabbit IgG, ab6721, 1:5000; Abcam) was added and then incubated for 1 hr and rinsed in TBST for 3 times. After visualization with Enhanced Chemiluminescence Plus (ECL Plus) Detection System (Thermo Scientific), the immunoreactive proteins were processed by Lab Works 4.5 software to detect the integral optical density value. GAPDH was used to compare with the proteins in grey density value.

### Cell transfection

MiR‐217 mimics (HMI0383) and miR‐217 inhibitor (HLTUD0383) were purchased from Sigma‐Aldrich (St. Louis, MO, USA). Si‐LINC01614 (sense1: 5′‐GCUGGAAGCAUUUCGUAAU‐3′, anti‐sense1: 5′‐AUUACGAAAUGCUUCCAGC‐3′; sense2: 5′‐GCCCACCTCAAATCCTGAA‐3′, anti‐sense2: 5′‐GCCCTCCTAAATCCACGAA‐3′) and si‐FOXP1 (sense1: 5′‐GCAGUUAGAGCUACAGCUU‐3′, anti‐sense1: 5′‐AAGCUGUAGCUCUAACUGC‐3′; sense2: 5′‐CCTTCCAAGTCCTCCCTAA‐3′, anti‐sense2: 5′‐CCTAACCTGCTCCCTCTAA‐3′) were obtained from Sangon Biotech, Shanghai, China. The most suitable transfection concentration for si‐LINC01614 and si‐FOXP1 was shown in Figure S1. Cells were grouped according to the following patterns: (*i*) mock group; (*ii*) negative control (NC) group: cells transfected with neo‐vector; (*iii*) miR‐217 mimics group: cells transfected with miR‐217 mimics; (*iv*) miR‐217 inhibitor group: cells transfected with miR‐217 inhibitor; (*v*) siRNA1 group: cells transfected with siRNA1‐LINC01614; (*vi*) siRNA2 group: cells transfected with siRNA2‐LINC01614; (*vii*) siRNAa group: cells transfected with si RNA1‐*FOXP1*; (*viii*) siRNAb group: cells transfected with siRNA2‐*FOXP1*; (*ix*) Mix‐1 group: cells transfected with siRNA1‐LINC01614+miR‐217 inhibitor; (*x*) Mix‐2 group: cells transfected with siRNAa‐*FOXP1*+miR‐217 inhibitor. Cells were transfected using Lipofectamine 2000 (Invitrogen) in accordance with the manufacturer's directions.

### Colony formation assay

Cells in the period of logarithmic phase were isolated by pancreatic enzyme digestion and then incubated in RPMI1640 medium. Afterwards, cells were inoculated in 6‐well plates at a density of 1 × 10^3^cells/well. Cells were shaken evenly and then put into an incubator supplemented with 5% CO_2_ at 37°C. Cultivation was terminated at the sight of visible cell clones. After being washed with FBS and dehydrated, cells were fixed with methanol and stained with violet. Last, staining fluid was removed and cells were dried. Five fields were randomly selected, and cells were counted with a microscope. All experiments were practised three times.

### MTT assay

To prepare a single cell suspension, cells were cultured in a medium with 10% FBS and then plated in 96‐well plates at a density of 2 × 10^3^cells/well. Then, 10 μl of MTT reagent (5 mg/ml, Sigma‐Aldrich) was added to each well and incubated for 4 hrs at 37°C. After culturing termination, supernatant in each well was separated and discarded (suspension cell need to be centrifuged first). Samples in each well were dissolved in 100 μl DMSO, and the absorbance was recorded at 490 nm using ELISA plate reader.

### Dual‐luciferase reporter gene assay

Cells were inoculated in 24‐well plates at a density of 1 × 10^5^cells/well, and when they entered logarithmic phase, they were transfected with different constructs as follows. 3′‐UTR of wild‐type LINC01614, mutant LINC01614, wild‐type *FOXP1* and mutant *FOXP1* were generated by PCR. Target sequence (LINC01614‐wt, LINC01614‐mut, *FOXP1*‐wt and *FOXP1*‐mut) was cloned to restriction enzyme sites (XbaI and FseI) of pGL3‐Basic vector (Promega, Madison, WI, USA). NCI‐H1395 and NCI‐H1975 cells were seeded into 24‐well plates, after which they were transfected with pGL3‐LINC01614‐wt, pGL3‐LINC01614‐mut, pGL3‐*FOXP1*‐wt or pGL3‐*FOXP1*‐mut. Meanwhile, miR‐217 mimics or mimics NC were cotransfected with Lipofectamine 3000 transfection reagent (Life Technologies, Gaithersburg, MD, USA). Transfection efficiency was normalized by cotransfecting with Renilla luciferase vector pRL‐SV50 (Promega); 48 hrs after transfection, the Dual‐Luciferase Reporter Assay System (Promega) was used to measure the luciferase activities using a luminometer.

### Statistical analysis

Data comparison between two groups was carried out using Student's two‐tailed *t‐*test, while comparison among three or more groups was employed with one‐way analysis of variance (anova). All experiments were repeated at least three times. All statistical calculations were performed by the software package GraphPad Prism version 6.0 (GraphPad Prism, La Jolla, CA, USA). A value of *P* < 0.05 was considered statistically significant. All data are present as mean ± S.D.

## Results

### Down‐regulated LINC01614 inhibited LUAD progression

**Figure 1:**
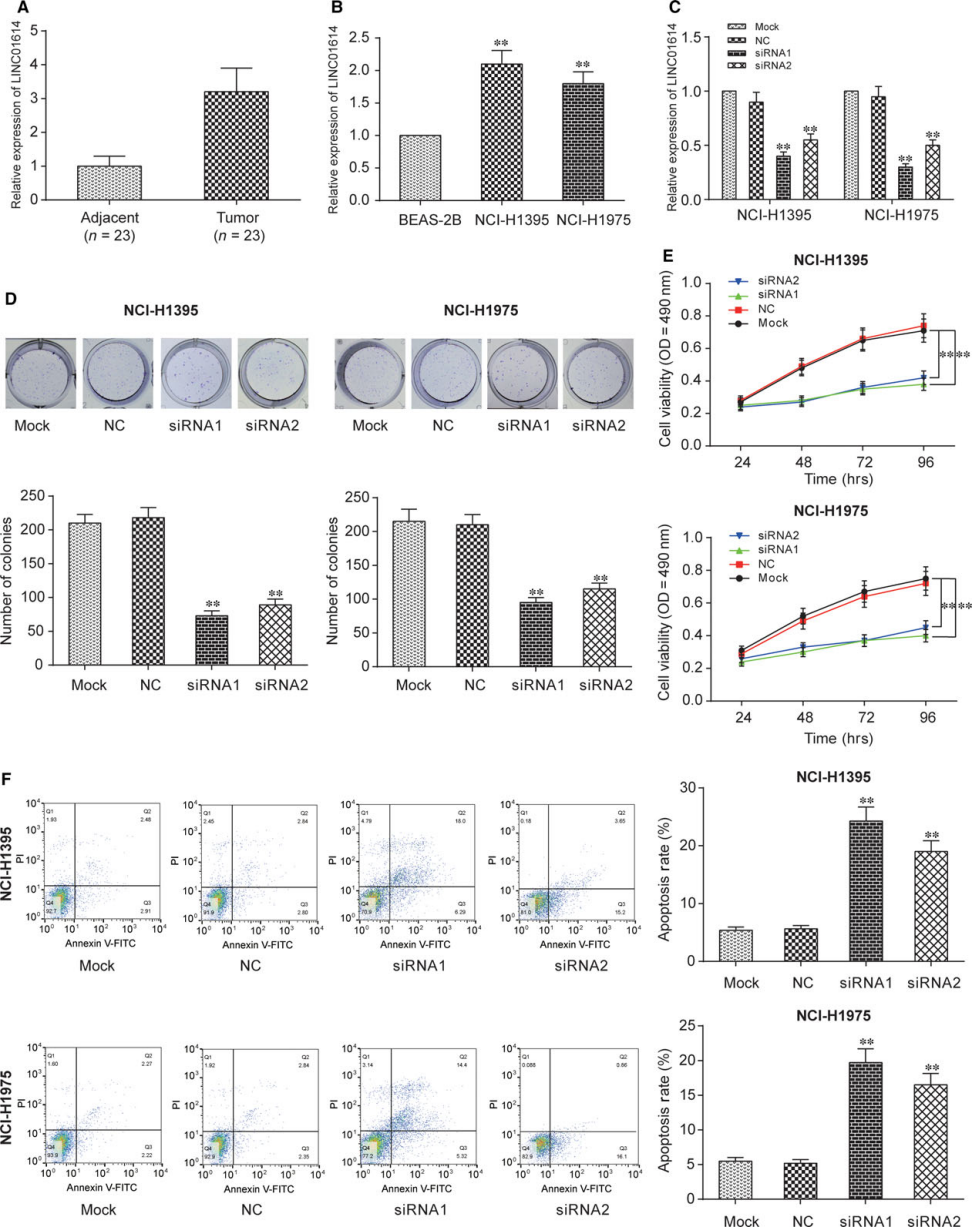
Down‐regulated LINC01614 inhibited the lung adenocarcinoma cells proliferation. **A**: The expression of LINC01614 was higher in LUAD tissues than in the adjacent normal tissue. **P < 0.01 *versus* adjacent. **B**: LINC01614 expression level was higher in LUAD cell lines than in the normal cell lines. **P < 0.01 *versus* BEAS‐2B. **C**: The expression of LINC01614 was low in cells which transfected with siRNA1 and siRNA2 of LINC01614. **P < 0.01 *versus* mock. **D**: The results of colony formation assay showed that the cell proliferation was inhibited when the LINC01614 expression level was inhibited. **P < 0.01 *versus* mock. **E**: The results of MTT assay showed similar results as the colony formation assay. **P < 0.01 *versus* mock. **F**: Apoptosis rate was higher in the siRNA1 and siRNA2 group than in the mock group. **P < 0.01 *versus* mock.

Twenty‐three cancerous and adjacent noncancerous tissues were used to identify the expression level of LINC01614 in LUAD *via* qRT‐PCR. The analysis showed that LINC01614 was highly expressed in LUAD tissues compared to noncancerous tissues (Fig. 1A, *P* < 0.01). Additionally, the expression levels of LINC01614 in two LUAD cell lines (NCI‐H1395, NCI‐H1975) and normal pulmonary epithelial cells (BEAS‐2B) were detected (Fig. 1B), and qRT‐PCR results showed the LINC01614 were significantly up‐regulated in two LUAD cell lines compared to BEAS‐2B cells (*P <* 0.01). After being transfected with siRNA1 or siRNA2, LINC01614 expression was significantly down‐regulated and siRNA1 displayed a strong inhibition effect (Fig. 1C, *P <* 0.01). Figure S1 showed that the most suitable transfection concentration for siRNAs was 50 nM considering transfection and economy efficiency. Then, we observed the variation of cell proliferation through colony formation assay and MTT assay, the results of which indicated that the cell proliferation was attenuated when the LINC01614 was inhibited (Fig. 1D and E, *P <* 0.01). Apoptosis analysis suggested that apoptosis rate was higher in siRNA1‐ and siRNA2‐transfected LINC01614 groups than in the mock groups (Fig. 1F, *P <* 0.01). Taken together, we revealed that LINC01614 knockdown inhibited cell proliferation and promoted cell apoptosis in LUAD cells. Therefore, we verified that the LINC01614 contributed to growth and metastasis of LUAD.

### Inhibition of LINC01614 restrained the expression of *FOXP1*

**Figure 2:**
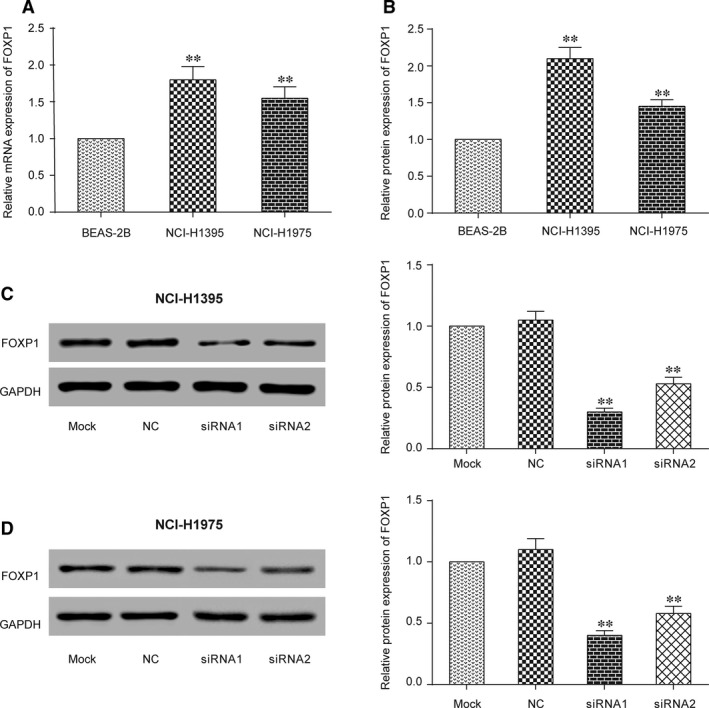
Inhibition of LINC01614 restrained the expression of *FOXP1*. **A**: qRT‐PCR results showed that *FOXP1* expression was higher in LUAD cell lines than in the normal cell lines. **P < 0.01 *versus* BEAS‐2B. **B**: Western blot results indicated similar results as qRT‐PCR. **P < 0.01 *versus* BEAS‐2B. **C**: The *FOXP1* protein decreased in siRNA1 and siRNA2 group in NCI‐H1395 cell lines. **P < 0.01 *versus* mock. **D**: The expression of *FOXP1* was decreased in siRNA1 and siRNA2 group in NCI‐H1975 cell lines. **P < 0.01 *versus* mock.

By qRT‐PCR and Western blot, we proved *FOXP1* was overexpressed in NCI‐H1395 and NCI‐H1975 cell lines compared to BEAS‐2B (Fig. 2A and B, *P* < 0.01). In both tumour cell lines, LINC01614 knockdown significantly inhibited *FOXP1* expression (Fig. 2C and D, *P* < 0.01). Therefore, we supposed that LINC01614 may impact cancer development by regulating *FOXP1*.

### Down‐regulation of *FOXP1* inhibited LUAD progression

**Figure 3:**
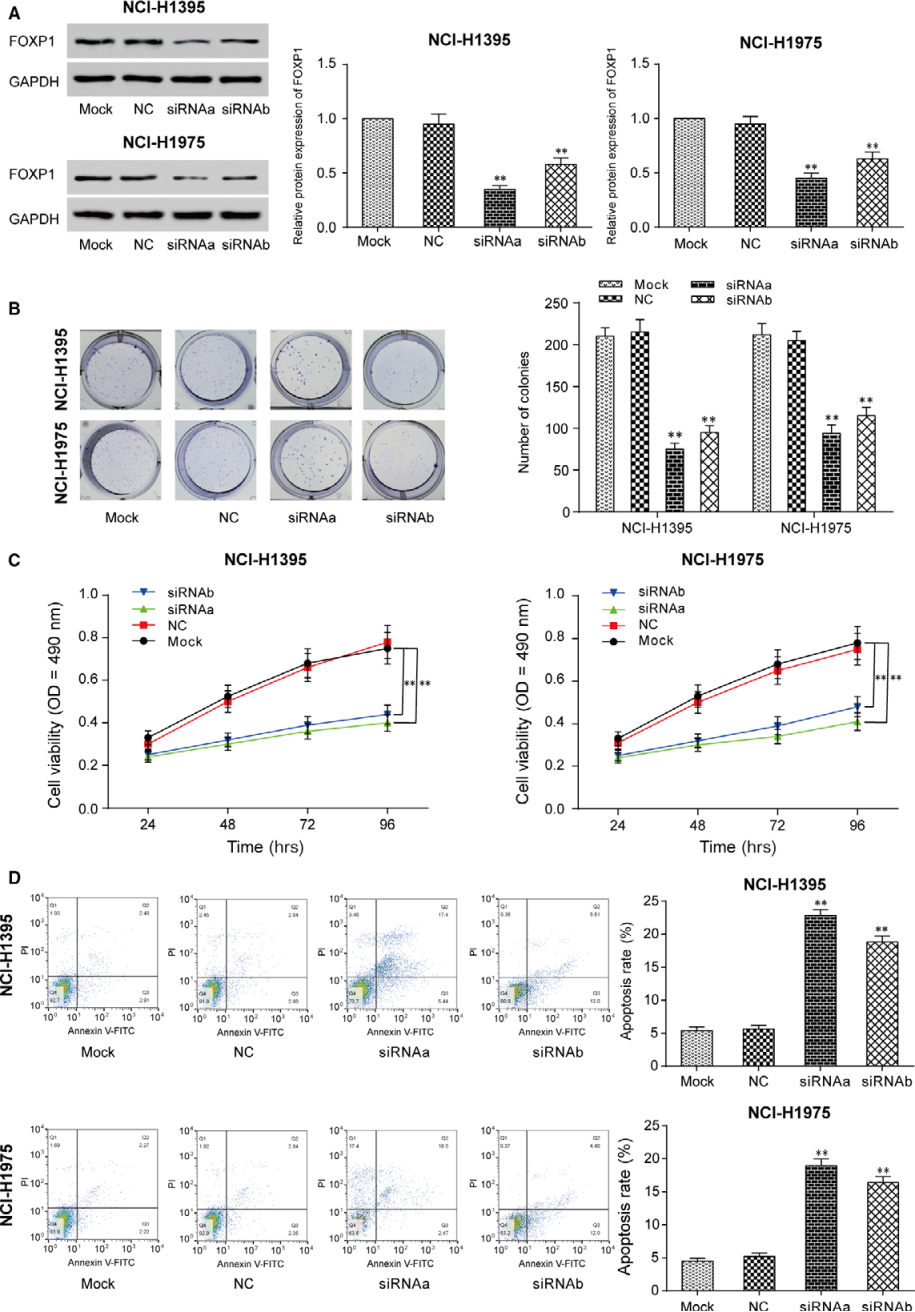
Down‐regulation of *FOXP1* inhibited cells proliferation. **A**: The protein expression of *FOXP1* was decreased in siRNAa and siRNAb of the *FOXP1* group. **P < 0.01 *versus* mock. **B**: Colony formation assays. The result of colony experiment on NCI‐H1395 and NCI‐H1975 which stably low expressed *FOXP1* for 2 weeks. **P < 0.01 *versus* mock. **C**: The result of MTT assay showed similar results as the colony formation assay **P < 0.01 *versus* mock. **D**: Apoptosis experiment. The result of apoptosis experiment shows that inhibition of *FOXP1* promoted the apoptosis of lung adenocarcinoma cells. **P < 0.01 *versus* mock.

SiRNAa and siRNAb of *FOXP1* were transfected into NCI‐H1395 and NCI‐H1975 cells to down‐regulate *FOXP1* expressions. We optimized the transfection concentration of si‐*FOXP1* in 50 nM, which was shown in Figure S1. Then, we confirmed the expression of *FOXP1* was remarkably decreased by siRNAa or siRNAb (Fig. 3A, *P* < 0.01). Also, cell proliferation rate of NCI‐H1395 and NCI‐H1975 was found to be largely slashed in *FOXOP1*‐knockdown groups (Fig. 3B and C, *P <* 0.01). Furthermore, cell apoptosis assay revealed that down‐regulation of *FOXP1* could induce apoptosis in NCI‐H1395 and NCI‐H1975 cells (Fig. 3D). Therefore, a positive correlation between *FOXP1* expression and progression of LUAD was observed in all trials.

### LINC01614 and *FOXP1* 3′UTR are two targets of miR‐217

**Figure 4:**
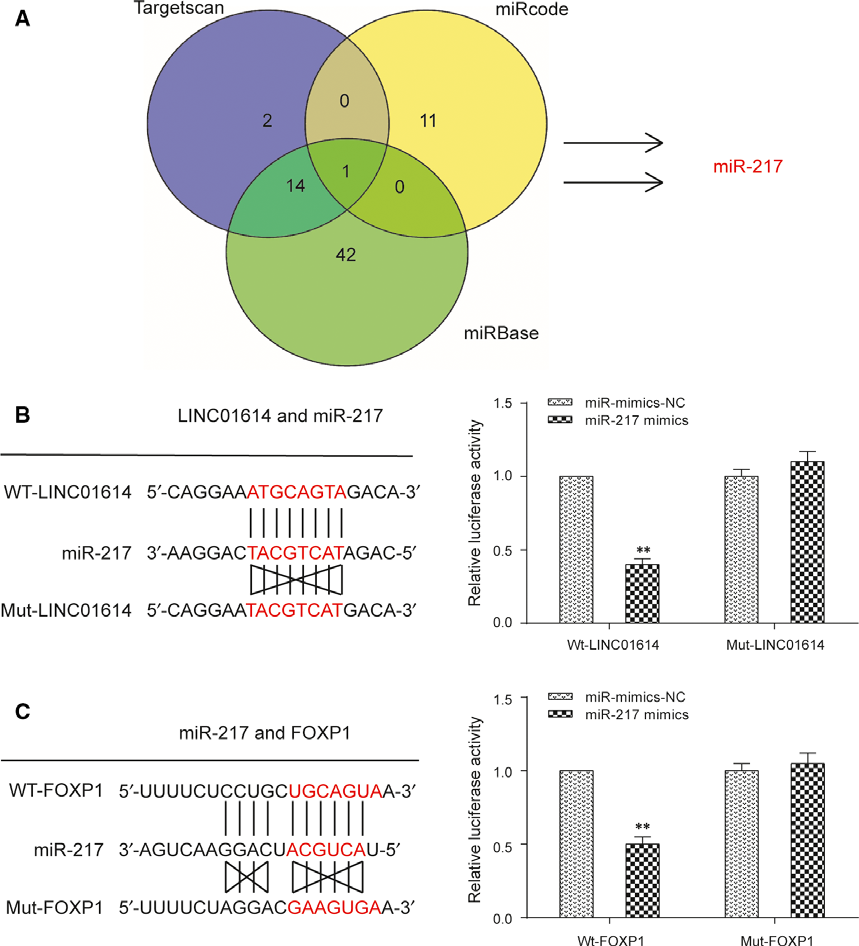
*FOXP1* and LINC01614 are two targets of miR‐217. **A**: Target prediction of miR‐217 by three different databases. **B**: Prediction of the LINC01614 binding sites to the miR‐217. The relative luciferase activity mediated by the reporter constructs harbouring the wild‐type (wt) or mutated LINC01614 upon transfection with miR‐217 mimics. **P < 0.01 *versus* NC. **C**: The relative luciferase activity mediated by the reporter constructs harbouring the wild‐type (wt) or mutated *FOXP1* upon transfection with miR‐217 mimics. **P < 0.01 *versus* NC.

TargetScan (http://www.targetscan.org/vert_71/), miRBase (http://www.mirbase.org/) and MiRcode (http://www.mircode.org/) databases were employed to search the complementary sequences among lncRNAs, miRNAs and mRNAs. Fortunately, we found miR‐217 might be a target for both LINC01614 and *FOXP1* (Fig. 4A), which meant LINC01614 could potentially sponge miR‐217 and miR‐217 directly bound to *FOXP1* 3′UTR. After cotransfecting luciferase‐reporting plasmids containing either LINC01614 wild‐type, LINC01614‐mutated sequence, *FOXP1* wild‐type 3′UTR or *FOXP1*‐mutated type and miR‐217 mimics or control sequence, the luciferase activity was determined (Fig. 4B). The relevant results shown in Figure 4C supported that miR‐217 could bind to both LINC01614 and *FOXP1* 3′UTR.

### Si‐LINC01614 suppresses *FOXP1 via* the promotion of miR‐217

**Figure 5:**
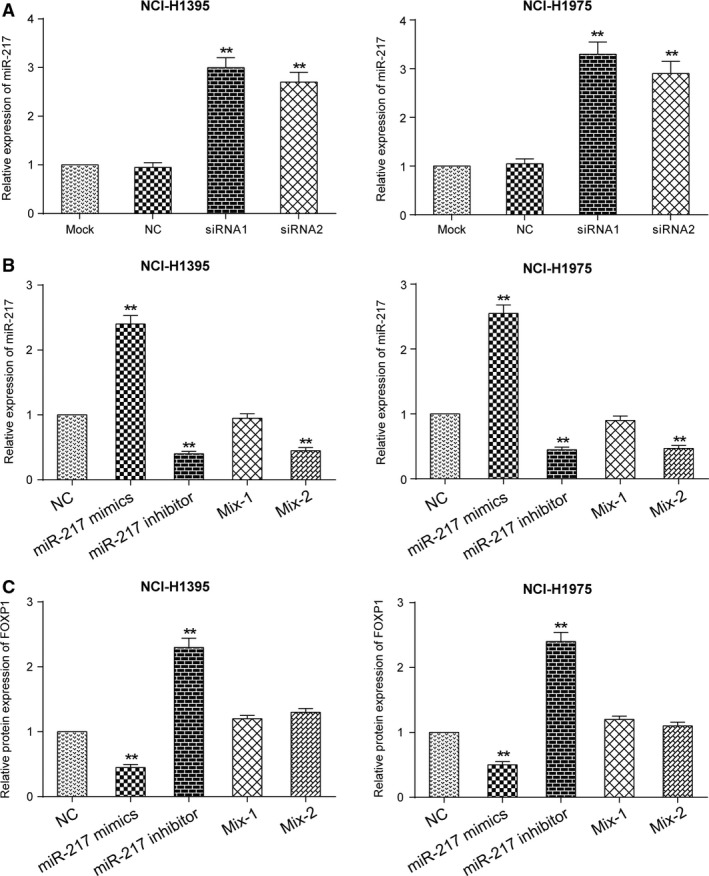
Si‐LINC01614 suppresses FOXP1 *via* the promotion of miR‐217 **A**: The expression of miR‐217 was decreased in the si‐LINC01614 group. **P < 0.01 *versus* mock. **B**: The miR‐217 expression in five groups, which indicated a successful transfection of miR‐217 mimics and miR‐217 inhibitor. **P < 0.01 *versus* NC. **C**: Effects of miR‐217 mimics or inhibitor on the expression of *FOXP1*. The expression of *FOXP1* was negatively correlated with the expression of miR‐217. **P < 0.01 *versus* NC.

qRT‐PCR showed that a negative correlation existed between the expression of LINC01614 and miR‐217 (Fig. 5A, *P <* 0.01). After being transfected with NC, miR‐217 mimics, miR‐217 inhibitor, miR‐217 inhibitor+siRNA1‐LINC01614 (Mix‐1), miR‐217 inhibitor+ siRNAa‐*FOXP1*(Mix‐2) in NCI‐H1395 and NCI‐H1975 cells, expression levels of miR‐217 and *FOXP1* were detected *via* qRT‐PCR and Western blot (Fig. 5B and C). Results suggested that the expression level of *FOXP1* decreased when miR‐217 was overexpressed, and reversely, the expression level of *FOXP1* rose when miR‐217 was suppressed (*P <* 0.01). Meanwhile, the expression variation of miR‐217 and *FOXP1* was not significant in Mix groups.

### LINC01614 knockdown inhibited cell proliferation through miR‐217/*FOXP1* axis

**Figure 6:**
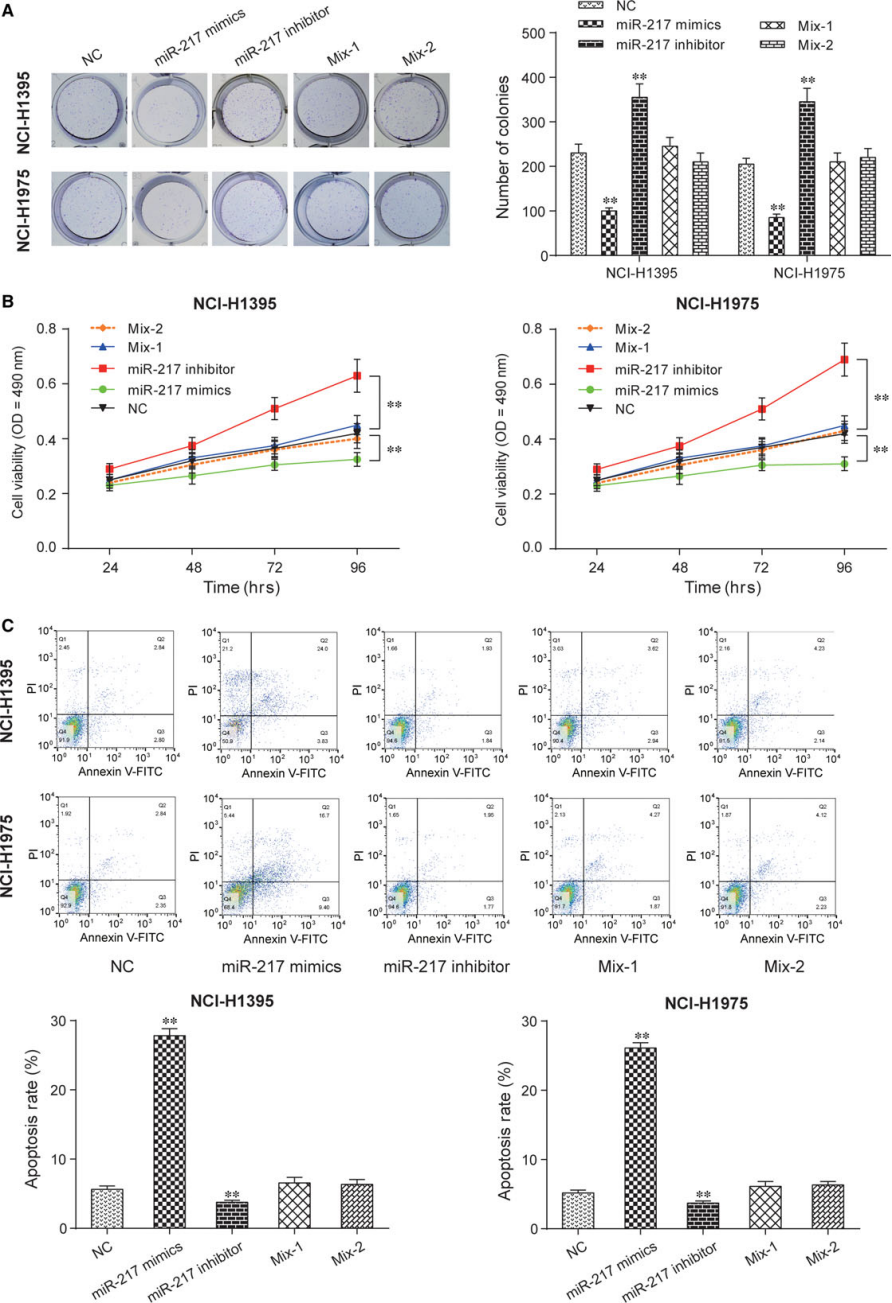
LINC01614 knockdown inhibited cell proliferation through miR‐217/*FOXP1* axis **A**: Colony formation assays. NCI‐H1395 and NCI‐H1975 were grown and transfected with miR‐217 mimics, miR‐217‐inhibition, miR‐217 inhibitor+si‐LINC01614, miR‐217 inhibitor+si‐*FOXP1* and then subjected to cell proliferation assay. **P < 0.01 *versus* NC. **B**: MTT assay witnessed the anti‐tumour effects of miR‐217 inhibitor. **P < 0.01 *versus* NC. **C**: The apoptosis was higher in miR‐217 mimics group than in NC group and lower in miR‐217 inhibitor groups than in the NC group. **P < 0.01 *versus* NC.

To clarify the LINC01614/miR‐217/*FOXP1* signalling axis, NCI‐H1395 and NCI‐H1975 were separately transfected with miR‐217 mimics, miR‐217‐inhibitor, miR‐217 inhibitor and siRNA1‐LINC01614 (Mix‐1) or miR‐217 inhibitor and siRNAa‐*FOXP1* (Mix‐2); 48 hrs after the transfection, cells were harvested and then subjected to cell proliferation assay. The results revealed that miR‐217 mimics significantly inhibited cell, whereas miR‐217 inhibitor effectively promoted colony formations (Fig. 6A and B). As Figure 6C showed, the apoptosis rates were dramatically increased in miR‐217 mimics groups in both NCI‐H1395 and NCI‐H1975 cells. Collectively, these data showed LINC01614, which suppressed miR‐217 and activated *FOXP1*, and then promote cell proliferation.

## Discussion

Mammalian genomes are confirmed to encode numerous lncRNAs, which used to be regarded as ‘junk’ or ‘transcription noise’. At present, there is growing evidence confirming that many lncRNAs are playing a very important role in bioprocesses 32, 33. Accommodative dysfunction of lncRNAs has been certified to exist in various diseases 34, 35, 36. Those lncRNAs could also provide more particular knowledge of cancer mechanism 37. Meanwhile, the majority of those cancer‐associated lncRNAs have been proved to be effective biomarkers 37, 38. However, deeper functions of tumour‐related lncRNAs remain to be unveiled. White *et al*. 4 has reported various lncRNAs related to LUAD *via* RNA‐seq data analysis. In the current study, we found a novel lncRNA, LINC01614, was up‐regulated among all LUAD tissues and cells. Moreover, inhibition of LINC01614 in NCI‐H1395 and NCI‐H1975 cells significantly decreased the cell proliferation, migration and invasion while markedly enhanced cell apoptosis. Thus, we came to the conclusion that down‐regulated LINC01614 inhibited the cell proliferation in LUAD.

It was approved by dozens of researches that *FOXP1* may be a very valuable marker for cancer patients’ prognosis. However, the expression patterns of *FOXP1* vary in different types of cancers. It was confirmed by Banham *et al*. 39 that complete loss of *FOXP1* protein was inhibited the progression of both six SCLCs and six NSCLCs. Simultaneously, Kyoko *et al*. have reported that reduced expressions of *FOXP1* may play key a role in the development of LUADs in rats 40. Intriguingly, by qRT‐PCR and Western blot, we identified that *FOXP1* was overexpressed in LUAD, which was in accordance with the research conducted by Feng *et al*. 28. We further discovered that down‐regulation of *FOXP1* inhibited cell progression of LUAD. Moreover, a positive correlation between *FOXP1* and LINC01614 expression can be drawn based on our results.

These results clued the underlying molecular mechanism in the carcinogenesis and metastasis of LUAD. According to the ceRNA hypothesis, lncRNAs may elicit their biological effects through their ability to act as endogenous decoys for miRNAs. Such activity would affect the ability of miRNAs to bind their targets 41. The present study showed that LINC01614 could regulate *FOXP1 via* miRNA. Through *in silico* analysis, we found miR‐217 could be a promising target of LINC01614 and *FOXP1*, which was then confirmed *via* luciferase assay. To further confirm the relationship among LINC01614, miR‐217 and *FOXP1*, we conducted the qRT‐PCR and Western blot experiments and verified that si‐LINC01614 suppresses *FOXP1 via* the promotion of miR‐217. In the end, cell proliferation assay revealed that LINC01614 knockdown significantly inhibited cell proliferation through miR‐217/*FOXP1* axis and vice versa. Apoptosis assay also showed that LINC01614 functioned as an inhibitor of miR‐217 and that it could increase the expression levels of the miR‐217 target gene, *FOXP1*, to promote cell proliferation.

Our study provides novel insights into the role of LINC01614 in cancer and may aid in the development of diagnostic and therapeutic tools for LUAD treatment. As no research about the LINC01614 function in diseases has been performed so far, our study is the first one that demonstrates the biological function of LINC01614 in LUAD. Further studies with a larger pool of samples should be conducted to verify the prognostic value of LINC01614 in LUAD. Meanwhile, further experiments such as whether FOXP1 does indeed act as a tumour suppressor in LUAD are in need.

## Funding source

None.

## Conflict of interests

The authors declare no conflict of interests.

## Supporting information


**Figure S1** The most suitable transfection concentration for si‐LINC01614 and si‐FOXP1 in NCI‐H1395 and NCI‐H1975 cells.
