# Supplementary figures and images for: Knockdown of LINC01614 inhibits lung adenocarcinoma cell progression by up‐regulating miR‐217 and down‐regulating FOXP1
Source: J Cell Mol Med. 2018 Jun 22;22(9):4034–44. doi: 10.1111/jcmm.13483 (PMC6111824; doi:10.1111/jcmm.13483)

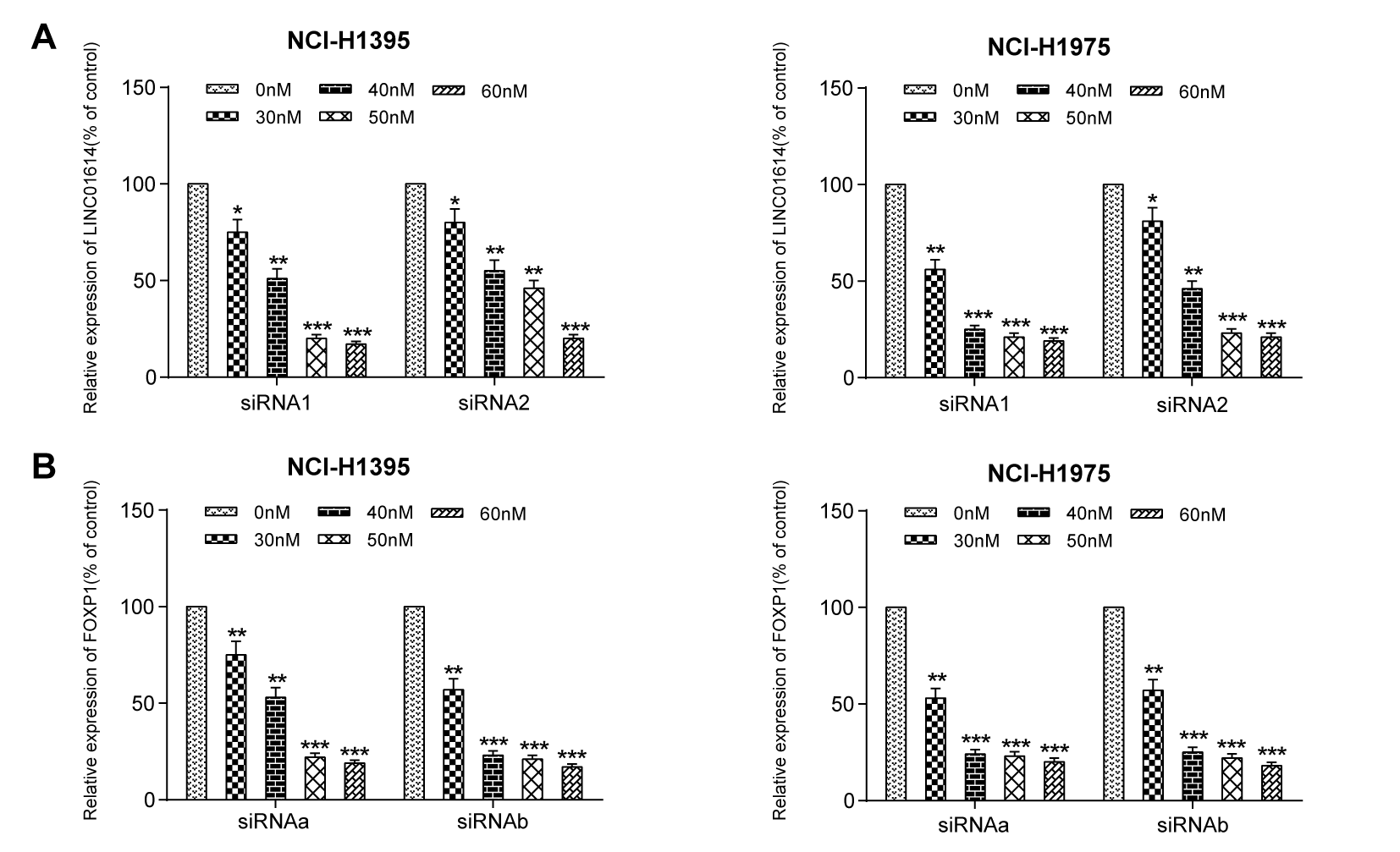

Supplement: Supplementary file 1 — Figure S1 The most suitable transfection concentration for si‐LINC01614 and si‐FOXP1 in NCI‐H1395 and NCI‐H1975 cells. [file JCMM-22-4034-s001.tif]
